# Extra and Intracellular Synthesis of Nickel Oxide Nanoparticles Mediated by Dead Fungal Biomass

**DOI:** 10.1371/journal.pone.0129799

**Published:** 2015-06-04

**Authors:** Marcia Regina Salvadori, Rômulo Augusto Ando, Cláudio Augusto Oller Nascimento, Benedito Corrêa

**Affiliations:** 1 Departamento de Microbiologia, Instituto de Ciências Biomédicas II, Universidade de São Paulo, São Paulo, São Paulo, Brazil; 2 Departamento de Química Fundamental, Instituto de Química, Universidade de São Paulo, São Paulo, São Paulo, Brazil; 3 Departamento de Engenharia Química, Politécnica, Universidade de São Paulo, São Paulo, São Paulo, Brazil; Institute for Materials Science, GERMANY

## Abstract

The use of dead biomass of the fungus *Hypocrea lixii* as a biological system is a new, effective and environmentally friendly bioprocess for the production and uptake of nickel oxide nanoparticles (NPs), which has become a promising field in nanobiotechnology. Dead biomass of the fungus was successfully used to convert nickel ions into nickel oxide NPs in aqueous solution. These NPs accumulated intracellularly and extracellularly on the cell wall surface through biosorption. The average size, morphology and location of the NPs were characterized by transmission electron microscopy, high-resolution transmission electron microscopy, scanning electron microscopy, and energy dispersive X-ray spectroscopy. The NPs were mainly spherical and extra and intracellular NPs had an average size of 3.8 nm and 1.25 nm, respectively. X-ray photoelectron spectroscopy analysis confirmed the formation of nickel oxide NPs. Infrared spectroscopy detected the presence of functional amide groups, which are probable involved in particle binding to the biomass. The production of the NPs by dead biomass was analyzed by determining physicochemical parameters and equilibrium concentrations. The present study opens new perspectives for the biosynthesis of nanomaterials, which could become a potential biosorbent for the removal of toxic metals from polluted sites.

## Introduction

Nanotechnology involves the manipulation and production of NPs with new properties, which differ significantly from their corresponding bulk solid-state material. According to the literature, these differences are due to effects such as quantum size effect, surface effect, and macroscopic quantum tunneling [1], [2]. Recently, nickel oxide NPs have attracted wide interest due to their applications in magnetic [3], electronic [4], optical [5], gas sensors [6], electrochemical films, photo electronic devices [7], catalysis, battery electrodes [8], and others. Metal NPs are synthesized by different physical and chemical methods. However, the importance of biological synthesis is being emphasized globally since chemical methods are capital and energy intensive, toxic, and have low yield [9].

The application of the highly structured physical and biosynthetic activities of microbial cells to the synthesis of nanosized materials has recently emerged as a novel approach to the synthesis of metal NPs [10]. New alternatives for the synthesis of metal NPs using bacteria, fungi and yeast are currently being explored [11]. Fungi have several advantages over other microorganisms for NPs synthesis since most species are easy to handle, require simple nutrients, and possess a high cell wall-binding capacity and high intracellular metal uptake capabilities [12], [13].

Contamination of sediments and natural aquatic environments with toxic metals is a major environmental problem around the world [14–17]. The introduction of nickel into the environment has increased over the last decades as a result of industrial pollution, for example from mining and smelting activities [18]. The interactions between microorganisms and metals have been well documented, [19] and the ability of microorganisms to extract and/or accumulate metals is already used in biotechnological processes such as bioremediation through the biosorption of toxic metals such as nickel. However, the mechanisms underlying these processes have not been elucidated. Fungi are frequently used in bioremediation processes since they are able to adapt and grow under extreme conditions of pH, temperature and nutrient availability, as well as at high metal concentrations [20].

Hence, there is increasing interest to develop processes for the biosynthesis of nickel NPs as an alternative to chemical and physical methods. According to a literature review, several studies have investigated the biosynthesis of metal NPs using live biomass of fungi [21]. However, there are few reports using dead fungal biomass for the synthesis of metal NPs [22–25]. We recently described the use of dead biomass of *Hypocrea lixii* (*H*. *lixii*) isolated from the wastewater of a mine in the Amazon region for the production of metallic copper NPs [22]. Aiming at green chemistry and biological processes, we developed an environmentally friendly approach to the synthesis of nickel oxide NPs using dead biomass of the filamentous fungus *H*. *lixii*. This is the first study describing the extra and intracellular biosynthesis and uptake of nickel oxide NPs from aqueous solutions using dead biomass of *H*. *lixii*.

## Materials and Methods

### Ethics Statement

The company Vale S.A., owner of Sossego Mine, located in Canaã, Pará, in the Brazilian Amazon region, through the director of the Vale Technology Institute, Dr Luiz Eugenio Mello authorized the establishment and dissemination of the study featured in this research article, allowing the collection of material (water from pond of copper waste) supervised by company employees, whose material led to the isolation of the fungus under study. This field study did not involve manipulation of endangered or protected species by any government agency.

### Fungal growth and maintenance

The fungus *H*. *lixii* used in this study was isolated from sediment and water samples of a copper waste pond of the Sossego mine located in Canãa dos Carajás, Pará, Brazilian Amazonia region (06° 26’ S latitude and 50° 4’ W longitude) [22]. *H*. *lixii* was selected for use in this study based on the determination of the minimum inhibitory concentration (MIC) of nickel as described below. The fungus was maintained and activated on Sabouraud Dextrose Agar (SDA) (Oxoid, England) [26].

### Screening for nickel (II)-resistant fungi

Nickel resistance of the fungi were determined as the MIC by the spot plate method [27]. SDA plates containing different nickel concentrations (50 to 2000 mg L^-1^) were prepared and inocula of the tested fungi were spotted onto the metal and control plates (plate without metal). The plates were incubated at 25°C for at least 5 days. The MIC is defined as the lowest metal concentration that inhibits visible growth of the isolates.

### Biosorption for nickel oxide NPs synthesis

All chemicals were of analytical grade and were used without further purification. All dilutions were prepared in ultrapure water purified in a Milli-Q system (Millipore, Milford, MA). The nickel stock solution was prepared by dissolving NiCl_2_.6H_2_O (Carlo Erba, Milan, Italy) in double-deionized water. The working solutions were prepared by diluting this stock solution.

The three types of fungal biomass (live, dried and dead) of *H*. *lixii* were prepared according to Salvadori et al. [22]. The effect of pH (2–6), temperature (20–60°C), contact time (5–300 min), initial nickel concentration (50–500 mg L^-1^), and agitation rate (50–250 rpm) on the removal of nickel was analyzed. These experiments were optimized at the desired pH, temperature, metal concentration, contact time, agitation rate and biosorbent dose (0.15–1.25 g) using 45 mL of a 100 mg L^-1^ Ni (II) test solution in plastic flask. After the desired contact time, the Ni (II) solution was separated from the biomass by vacuum filtration through a Millipore membrane and the residual metal ion concentration was determined by inductively coupled plasma optical emission spectrometry. The efficiency (R) of metal removal was calculated using the equation:

$$R=(C_{i}-C_{e})/C_{i}.100$$

where C_i_ and C_e_ are the initial and equilibrium metal concentration, respectively. The metal uptake capacity, q_e_, was calculated using the equation:

$$q_{e}=V(C_{i}-C_{e})/M$$

where q_e_ (mg g^-1^) is the biosorption capacity of the biosorbent at any time, M (g) is the biomass dose, and V (L) is the volume of the solution.

### Sorption isotherm

The Langmuir equilibrium model [28] was used to fit the Ni (II) biosorption isotherm experimental data. The linearized Langmuir isotherm model according to equation:

$$C_{e}/q_{e}=1/(q_{m}.b)+C_{e}/q_{m}$$

where q_m_ is the monolayer sorption capacity of the sorbent (mg g^-1^), and b is the Langmuir sorption constant (L mg^-1^).

### Characterization of the extra and intracellular biosynthesis of nickel oxide NPs by dead biomass of *H*. *lixii*

In this study only dead biomass of *H*. *lixii* was used for the analysis of nickel oxide NPs production since it exhibited a high adsorption capacity of the nickel metal ion than live and dried biomass. Biosynthesis of nickel oxide NPs by dead biomass of *H*. *lixii* was investigated under the following conditions: contact time of 90 min, initial pH 4.0, temperature of 30°C, agitation speed of 150 rpm, 1.0 g of the biosorbent, and a solution containing 100 mg L^-1^ of nickel (II). The synthesized nickel oxide NPs were characterized by transmission electron microscopy (TEM) (JEOL-1010) operating at 100 kV, high-resolution transmission electron microscopy (HRTEM) (JEOL JEM2100 equipped with a LaB6 gun) operating at 200 kV, X-ray photoelectron spectroscopy (XPS) (UNI-SPECS UHV System), scanning electron microscopy (SEM) (JEOL 6460 LV) equipped with an energy dispersive spectrometer (EDS) and infrared vibrational spectroscopy FTIR (Bruker ALPHA) using an attenuated total reflectance accessory of single reflection (ATR with platinum-crystal diamond).

## Results and Discussion

Analysis of different nickel MIC (50–2000 mg L^-1^) showed that *Aspergillus aculeatus* exhibited the highest resistance to nickel among the strains isolated from sediment, as also reported in previous studies [25]. The fungus *H*. *lixii* isolated from water samples also showed high tolerance to nickel (1473 mg L^-1^) (Table 1). Since the objective of this study was to investigate the uptake and biosynthesis of NPs from aqueous medium, this fungus was selected for the study. Some fungi are recognized as hyperaccumulators of heavy metals due to their filamentous morphology and high content of cell wall material [29]. The hyphal wall of fungi has been found to be the primary site of metal ion accumulation owing to the presence of the acetamide group of chitin, amino and phosphate groups in nucleic acids, amino, amido, sulfhydryl and carboxy groups in proteins, and hydroxyls in polysaccharides [30].

**Table 1:** Minimum inhibitory concentrations obtained for the fungi isolated from water.

Fungi isolated	Nickel concentrations (mg L^-1^)
*Cladosporium cladosporioides*	71
*Curvularia brachyspora*	23
*Hypocrea lixii*	1473
*Myrothecium gramineum*	166
*Penicillium charlesii*	356
*Penicillium purpurogenum*	71
*Phoma putaminum*	No growth
*Phomopsis sp*	166
*Trichoderma tomentosum*	737

### Effect of physicochemical parameters on biosorption for nickel oxide NPs synthesis

**Fig 1:**
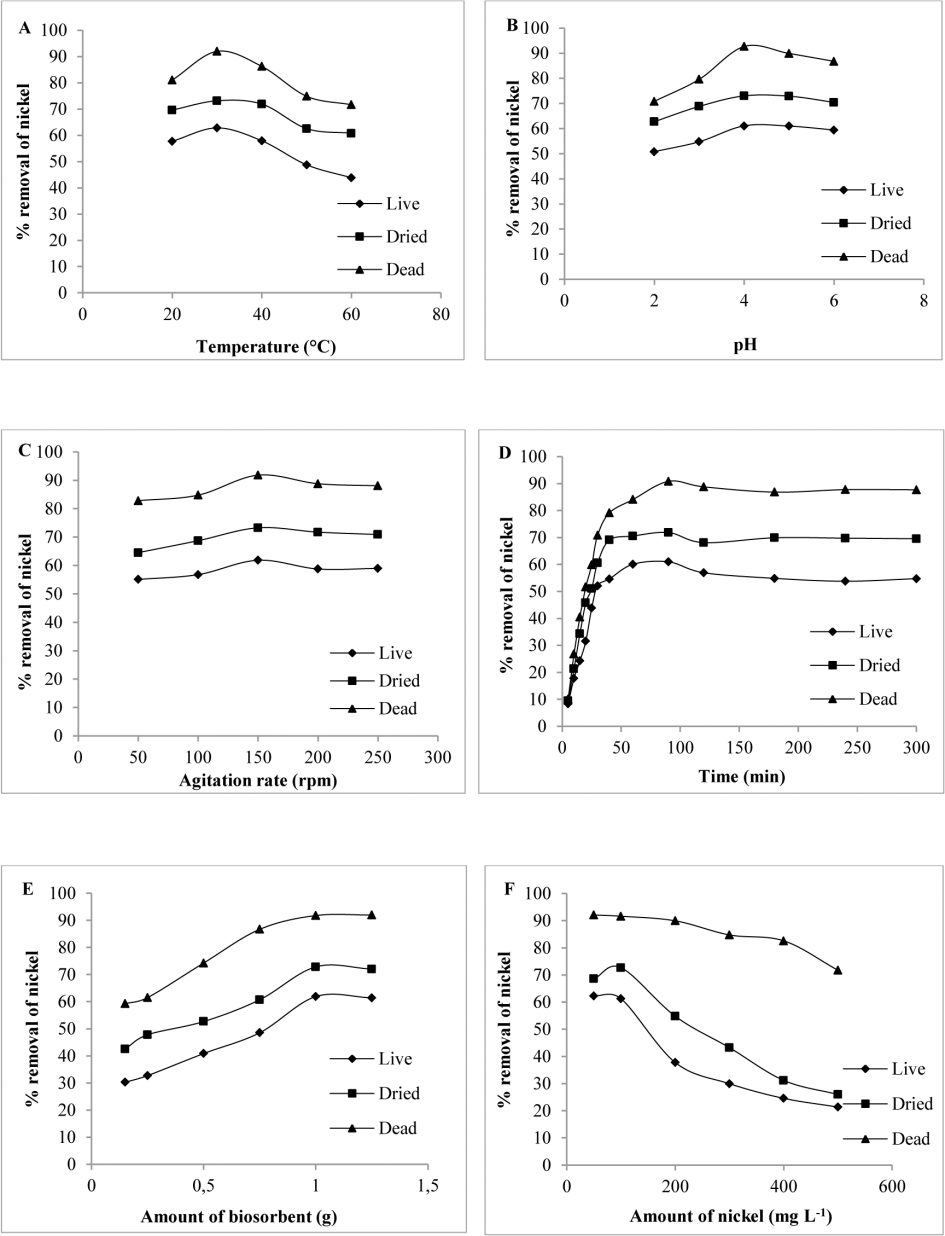
Biosorption studies. Biosorption properties of the three types of *H*. *lixii* biomass. (A) Effect of temperature. (B) Effect of pH. (C) Effect of agitation rate. (D) Effect of contact time. (E) Effect of the amount of biosorbent. (F) Effect of initial nickel concentration.

The biosorption properties of the three types of *H*. *lixii* biomass (live, dried and dead) were evaluated as a function of initial pH, temperature, agitation rate, biomass dose, contact time, and initial Ni (II) concentration to determine the satisfactory biosorption of Ni (II) ions. The present results showed that physicochemical parameters affect the removal of nickel from aqueous solution, in agreement with other studies [31–33]. The influence of temperature on nickel uptake is shown in Fig 1A. Maximum removal occurred at 30°C for the three types of biomass. According to the adsorption theory, adsorption decreases with increasing temperature as molecules absorbed earlier on a surface tend to desorb from the surface at higher temperatures [34]. The capacity of ionization of functional groups found on the biomass cell wall surface depends on the pH of the solution [35]. In this respect, maximum removal of nickel was observed at an initial pH of 4.0 for the three types of biomass (Fig 1B). Regarding agitation speed, optimum nickel removal was observed at 150 rpm for the three types of biomass (Fig 1C). Vortex phenomena occur at higher agitation speeds and the suspension is no longer homogeneous, a fact impairing metal removal [36]. The graph obtained for the three types of biomass exhibited sigmoid kinetics Fig 1D, characterizing an enzyme-catalyzed reaction. The results showed that this was a rapid process, with more than 90% of the NPs being formed by dead biomass within 90 min of the reaction. An important parameter to determine the sorbent-sorbate equilibrium of a system is the amount of biosorbent. As can be seen in Fig 1E, dead biomass was more efficient in removing nickel when compared to live and dried biomass, indicating that dead biomass possess a higher affinity for nickel [37]. The initial metal concentration provides an important driving force to overcome all mass transfer resistance of the metal between the aqueous and solid phases [38]. The percentage of nickel adsorption decreased with increasing metal concentration (50–500 mg L^-1^) for the three types of biomass (Fig 1F).

### Sorption isotherm

**Fig 2:**
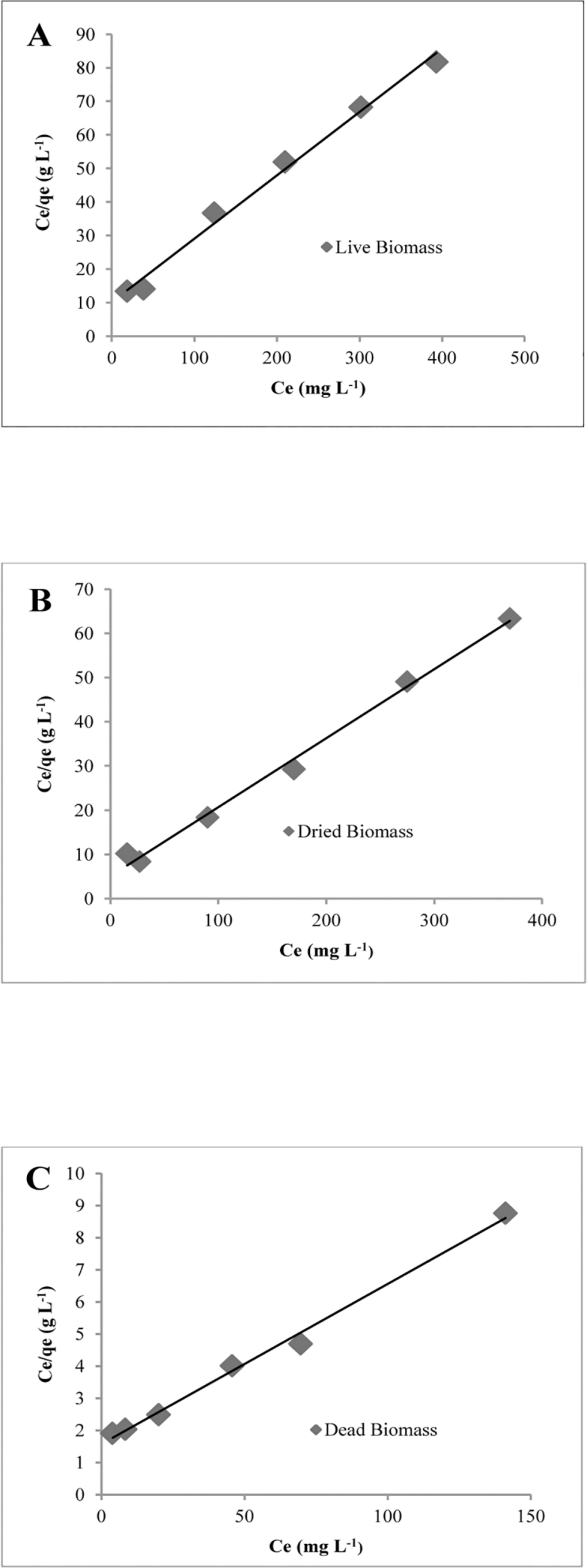
Equilibrium biosorption data. Langmuir plots for live (A), dried (B) and dead (C) biomass of *H*. *lixii*.

A maximum retention capacity of 5.3, 6.4 and 20.1 mg nickel g^-1^ was obtained for live, dried and dead biomass of *H*. *lixii*, respectively. Live and dried biomass is susceptible to the toxic effect of the metal concentration used in this study, a fact leading to low metal retention by these types of biomass. This is a disadvantage of the use of live and dried biomass over dead biomass, which has also been reported in other studies [39], [40]. The Langmuir isotherm equation was used to describe the biosorption of Ni (II) for the three types of biomass (Fig 2). Table 2 shows the isotherm constants, maximum loading capacity estimated with the Langmuir model, and regression coefficients. The maximum retention capacity of 20.1 mg nickel g^-1^ observed for dead *H*. *lixii* biomass was similar or higher than that reported for other living biosorbents, such as the marine algae *Ulva sp*. (17 mg g^-1^) [41], cone biomass of *Thuja orientalis* (12.4 mg g^-1^) [42], the bacteria *Escherichia coli* (6.9 mg g^-1^) [43], bacteria *Streptomyces coelicolor* (11.1 mg g^-1^) [44], and the fungi *Trichoderma harzianum* (11.77 mg g^-1^), *Rhizopus arrhizus* (9.28 mg g^-1^), *Aspergillus terreus* (7.86 mg g^-1^), *Aspergillus niger* (7.69 mg g^-1^), *Aspergillus flavus* (7.5 mg g^-1^), *Alternaria alternata* (7.37 mg g^-1^), *Cunninghamella echinulata* (4.69 mg g^-1^) [45] and others. There are also reports of other organisms used as dead biosorbents for the retention nickel, including *Mucor rouxii* [46], the marine algae *Sargassum vulgares*, *Sargassum fluitans* and *Galaxaura marginata* [47], *Fusarium flocciferum* [48], *Schizophyllum commune* [49] and others.

**Table 2:** Adsorption isotherm parameters for Ni (II) ions using live, dried and dead biomass of *H*. *lixii*.

	Langmuir model		
Type of biomass	q_m_(mg g^-1^)	b(L mg^-1^)	*R* ^*2*^
Live	5.3	0.018	0.991
Dried	6.4	0.030	0.993
Dead	20.1	0.031	0.994

### Extra and intracellular biosynthesis of nickel oxide NPs

**Fig 3:**
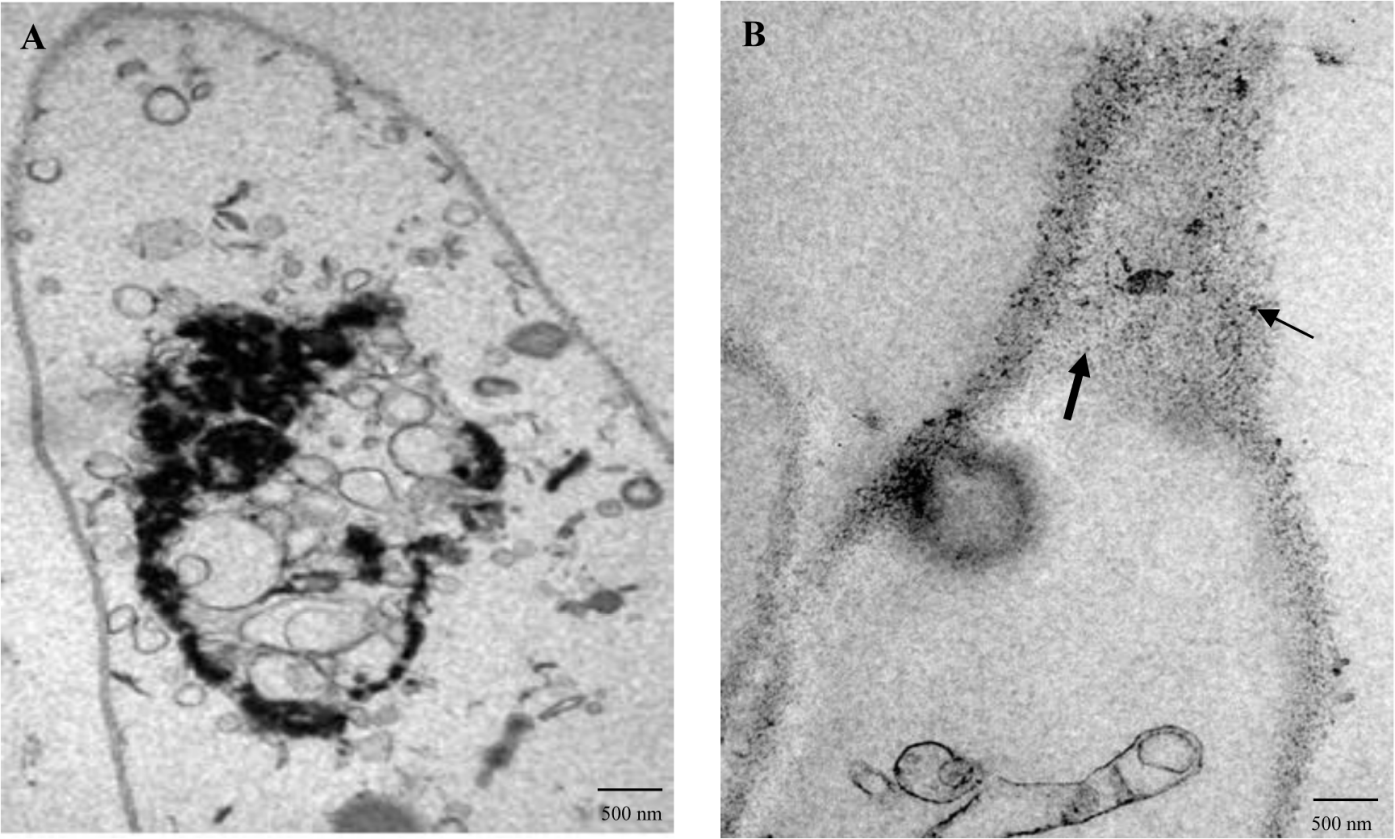
TEM photomicrograph of a section of dead *H*. *lixii* biomass. (A) Control (without nickel). (B) Section of the fungus showing extracellular (lighter arrow) and intracellular (darkest arrow) nickel oxide NPs.

**Fig 4:**
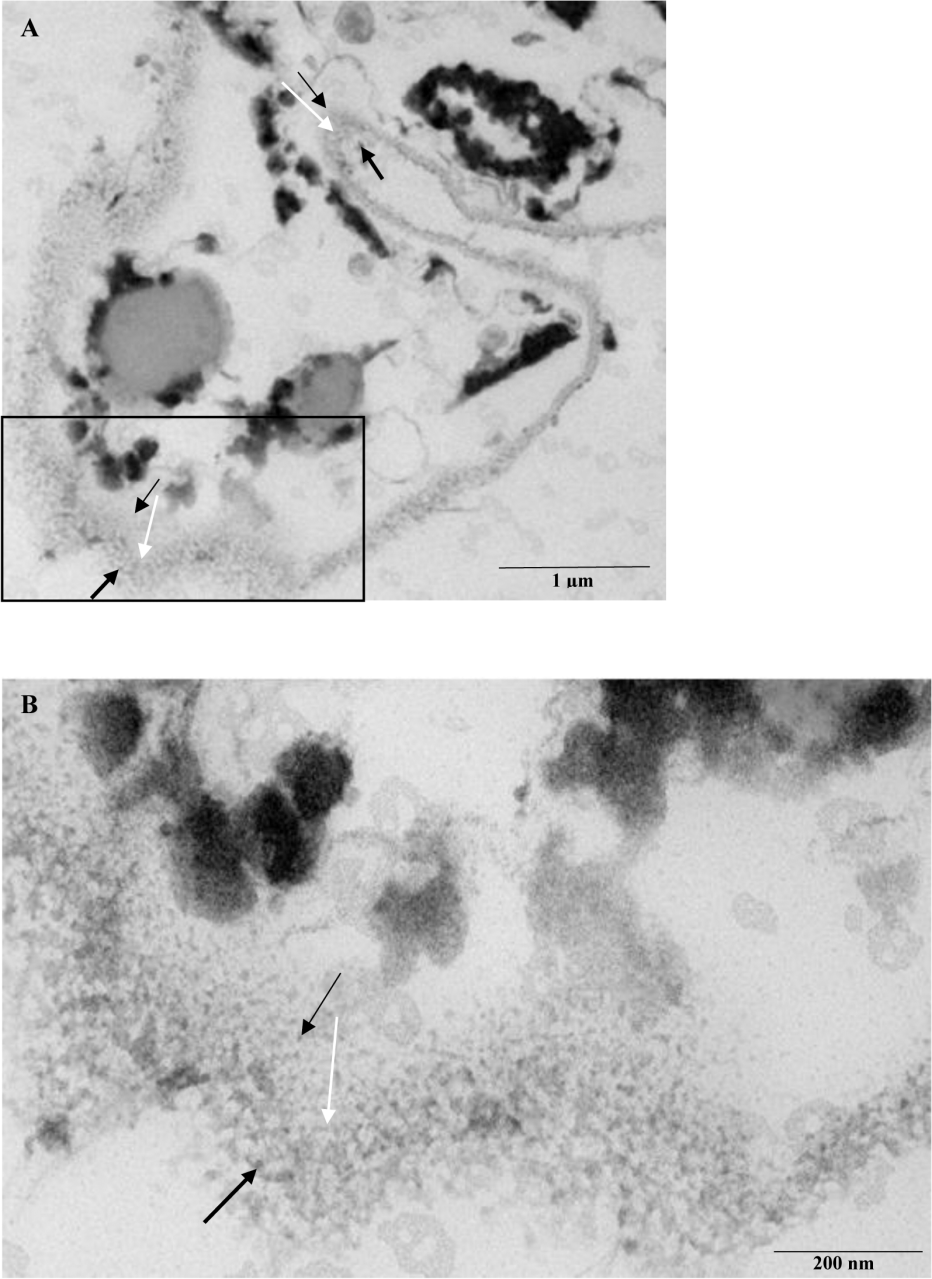
HRTEM photomicrograph of a section of dead *H*. *lixii* biomass. (A) Section of the fungus showing the cell wall (white arrow), extracellular (darkest black arrow) and intracellular (lighter black arrow) nickel oxide NPs. (B) Expanded section (square in A) of the fungus showing the cell wall (white arrow), extracellular (darkest black arrow) and intracellular (lighter black arrow) nickel oxide NPs.

The study of new biosynthetic routes of metal oxide NPs is important to identify alternatives to chemical and physical methods. After biosorption, a fraction of the dead *H*. *lixii* biomass loaded with NPs was examined by TEM and HRTEM (Figs 3 and 4). The TEM and HRTEM images provided information about the location of the nickel oxide NPs in the dead biomass and revealed the presence of extra and intracelular NPs. Fig 3A shows absence of NPs in the control. Ultrastructural changes included the shrinking of cytoplasmatic material in the control and in the biomass containing the metal NPs, which was probably the result of the autoclaving process (Fig 3A and 3B). TEM analysis revealed a predominantly spherical shape of the extra and intracellular nickel oxide NPs, which had an average diameter of 3.8 and 1.25 nm, respectively. The NPs formed inside the fungal cells were smaller than extracellularly produced NPs. The extra and intracellular synthesis of NPs of other metals has been reported, but those studies have used live fungal biomass [50].

**Fig 5:**
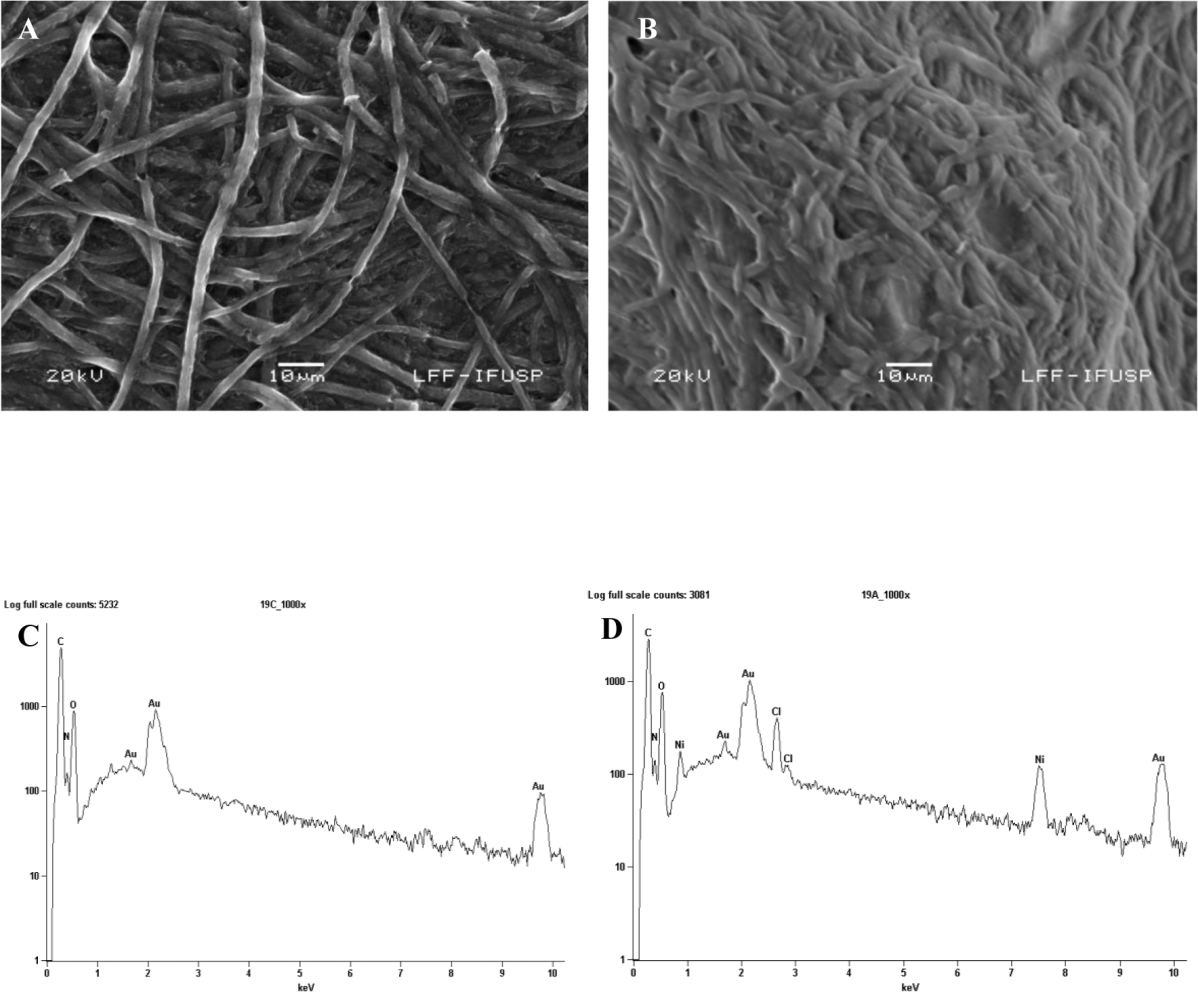
SEM images and EDS spectra of dead *H*. *lixii* biomass. Photomicrograph of the surface of dead *H*. *lixii* biomass before (A) and after (B) the adsorption of nickel ions. EDS spectra of dead *H*. *lixii* biomass before (C) and after (D) exposure to the metal.

**Fig 6:**
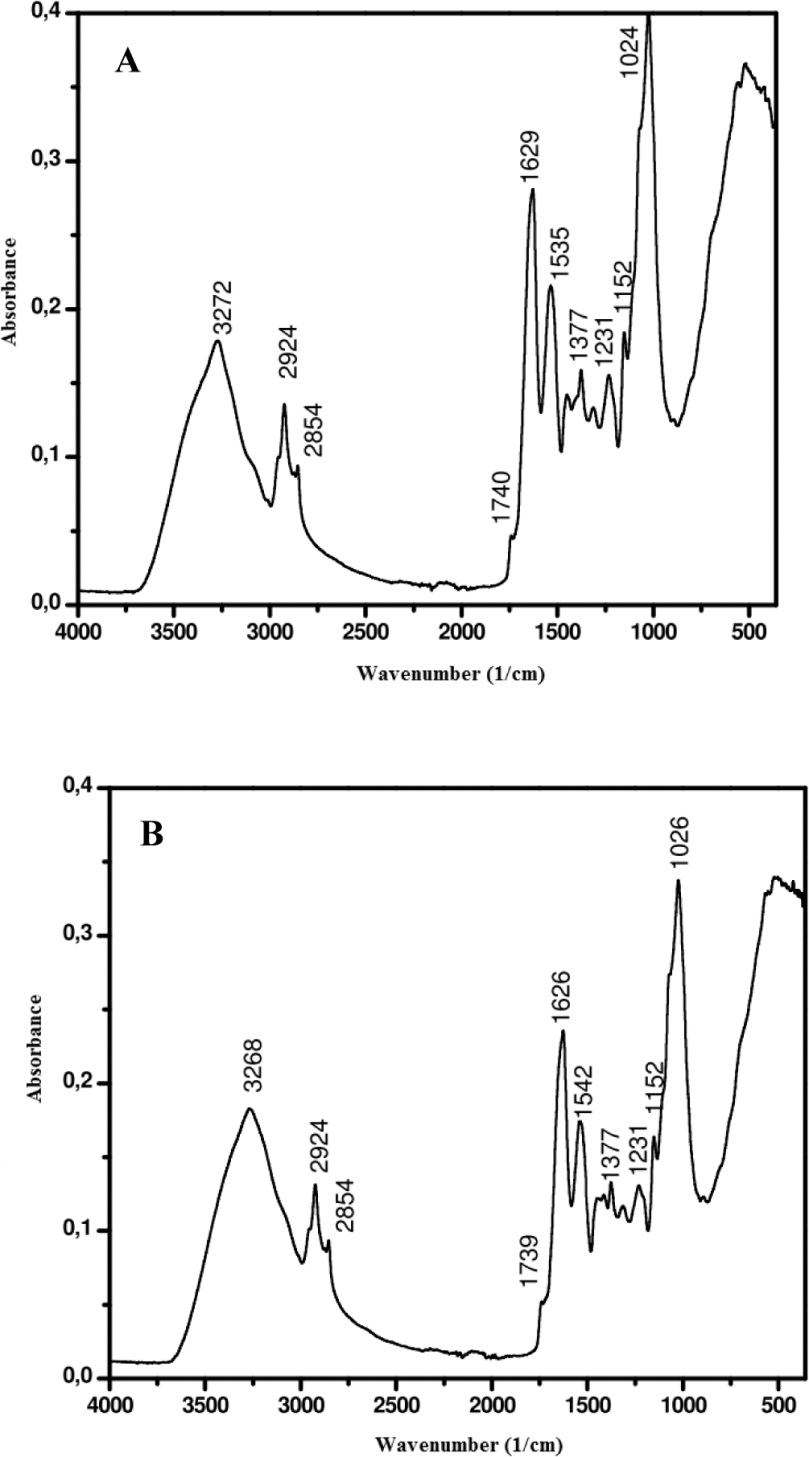
FTIR spectra of dead biomass of *H*. *lixii*. (A) Before and (B) After saturation with nickel.

**Fig 7:**
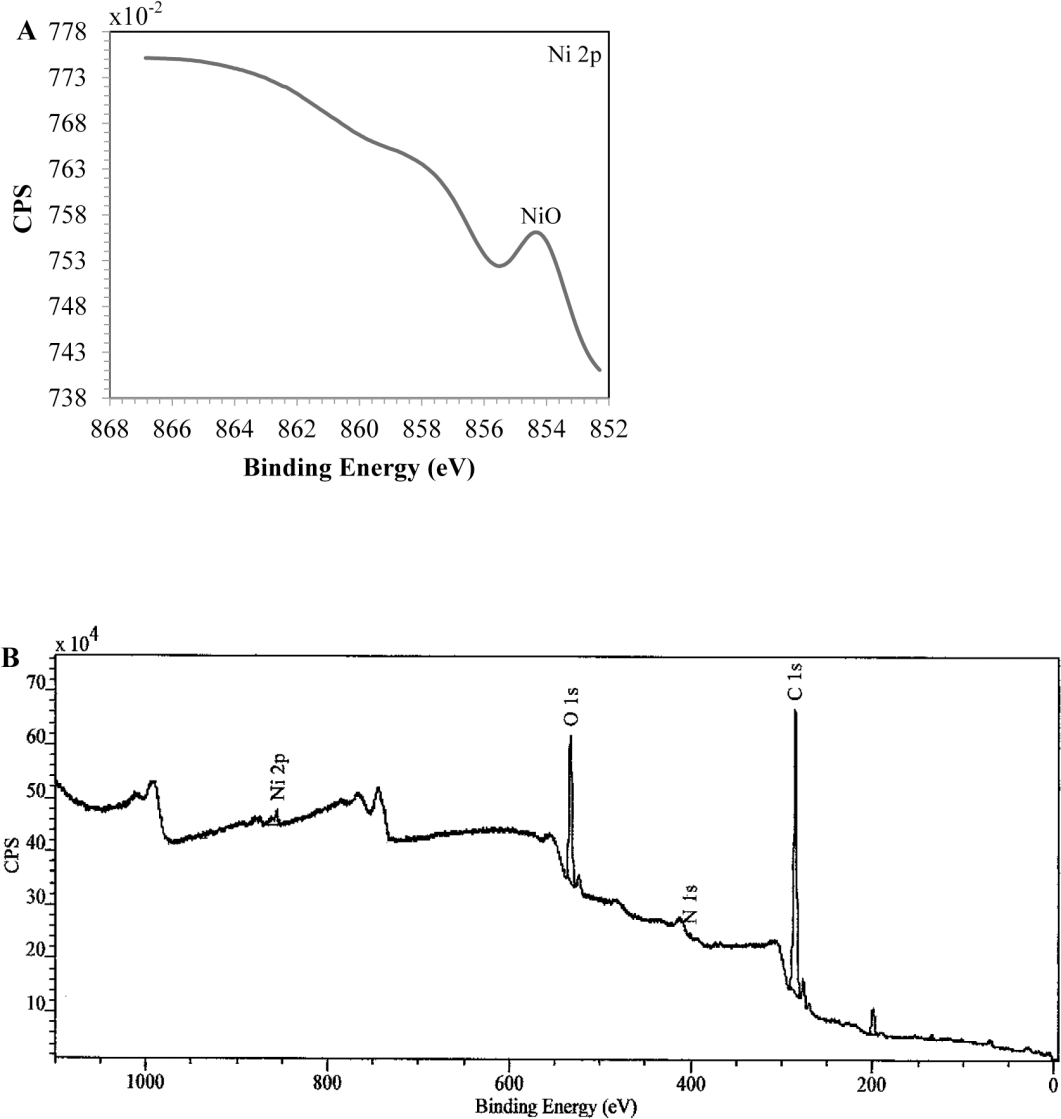
XPS spectra. (A) Ni 2p core level binding energy after synthesis of nickel oxide NPs by dead biomass of *H*. *lixii*. (B) Wide scan XPS spectrum after biosynthesis of nickel oxide NPs showing Ni 2p, O1s, N1s and C1s core level binding energies.

To confirm the presence of nickel in the fungus, the dead *H*. *lixii* biomass was analyzed by SEM-EDS. Comparison of the photomicrographs of dead biomass without nickel (Fig 5A) and impregnated with nickel (Fig 5B) revealed a subtle change in surface biomass. The EDS spectra recorded in the dead biomass area examined showed clear signals attributed to nickel (Fig 5C and 5D). C, N and O signals were also detected. These signals may be due to the presence of proteins and other biopolymers in the biomass that possibly act as capping material on the surface of the NPs. FTIR analysis of nickel oxide NPs illustrated a band at 1535 cm^-1^ that was shifted to 1542 cm^-1^ (Fig 6), assigned to N-H deformation of amide II linkages of polypeptides or proteins [51], corroborating the results of EDS analysis. Fig 7A shows the XPS spectrum of the Ni 2p core level after the biosynthesis of nickel oxide NPs by dead *H*. *lixii* biomass. The peak at 854.1 eV corresponds to the Ni 2p_3/2_ level and it is characteristic of NiO [52–54]. No peak was observed at 852.2 eV, which would be characteristic of the Ni 2p_3/2_ level of Ni (0) [55]. The wide scan XPS spectrum (Fig 7B) shows Ni 2p, O1s, N1s and C1s core level after synthesis of nickel oxide NPs by the fungus. The C1s core level had a peak at 285.0 eV, which could be attributed to aromatic and aliphatic amines of peptides/proteins probably bound to the nickel oxide NPs. In the O1s and N1s core level, binding energies were observed at 532.4 eV and 400.4 eV, respectively, confirming the possible presence of proteins involving the nickel oxide NPs, as also observed for copper nanoparticles [56].

The mechanisms and agents involved in the extra and intracellular biosynthesis of nickel oxide NPs by dead biomass of *H*. *lixii* are not fully understood. We propose a two-steps process that involves the reduction of Ni^2+^ ions and their subsequent extra and intracellular oxidation to NiO. The first step involves of the interaction between nickel ions and amide groups found in the fungal cell wall and then their bioreduction to metallic Ni, probably by enzymes present in the cell wall. Mostly, fungi are regarded as the organisms that produce NPs extracellularly because of their enormous secretory components, which are involved in the ion reduction and capping of NPs [21]. In fact, FTIR analysis of the peptides/proteins bound to the nickel oxide NPs suggests that they act as capping agents and prevent agglomeration, in agreement with the results obtained by EDS and XPS analysis. In the present study, the dead biomass probably used enzymes released from the cell during the autoclaving process and bound to the cell surface [22]. This extracellular mechanism of NPs synthesis has also been proposed for the synthesis of silver NPs [57]. The second step involves the oxidation of metallic NPs by water and oxygen present in the medium, due to the negative reduction potential of nickel.

The formation of an oxide passivation layer would be expected, but the XPS results showed that the NPs are formed solely by NiO, which may be a consequence of the very small size (high superficial area) of the NPs formed (1.25 nm and 3.8 nm for intracellular and extracellular NPs, respectively).

The intracellular synthesis of NPs is very similar to their extracellular synthesis: the Ni^2+^ ions interact with the fungal cell wall as a result of electrostatic interaction with enzymatic groups present in the mycelial cell wall and are then probably reduced by enzymes inside the cell wall, leading to the aggregation of nickel ions and formation of NPs. This enzyme-based pathway has also been proposed for the intracellular synthesis of silver NPs [58]. The difference in this case is that the formed NPs are smaller than extracellularly. In this case it was also observed the complete oxidation leading to the formation of nickel oxide NPs.

A possible process to separate extracellular nickel oxide NPs from the biomass is centrifugation [59], while intracellular NPs could be removed by ultrasound treatment of the biomass or by reaction with suitable detergents [60]. The advantage of the biological method described in this study compared to synthetic methods that comprise complex physical and chemical processes is the fact that it does not require the use of high temperatures, high pressures or large amounts of energy. Additionally, no toxic residues are produced which pose risks to the environment and human health [9]. The advantage of the use of dead biomass over live biomass is that, for binding to metal ions for NPs production, the latter depend on the availability of nutrients for their maintenance, environmental conditions and cell age and are susceptible to the toxic effect of the metals present in the medium [61]. Therefore, dead biomass is preferred to overcome these disadvantages.

## Conclusions

In this study, dead biomass of the fungus *H*. *lixii* was found to be an efficient tool for the extra and intracellular synthesis of nickel oxide NPs and for the uptake of toxic metal ions from aqueous solution. The dead biomass of the fungus played an important role by acting as a reducing agent to form metallic NPs, which are readily oxidized to nickel oxide in the medium. The dead biomass also acts as a stabilizer during the synthesis of nickel oxide NPs and could therefore be used for the uptake of nickel ions during bioremediation processes. The ability of dead fungal biomass to synthesize NPs is highly promising for the green sustainable production of nano-metals and increases its widespread application as a natural strategy. Obviously, the elucidation of the exact mechanisms involved in the extra and intracellular synthesis of metal NPs using dead biomass continues to be a major scientific challenge.
